# Case Report: Novel TRPM6 Mutations Cause Hereditary Hypomagnesemia With Secondary Hypocalcemia in a Chinese Family and a Literature Review

**DOI:** 10.3389/fped.2022.912524

**Published:** 2022-07-12

**Authors:** Yiran Han, Yajuan Zhao, Hua Wang, Liang Huo

**Affiliations:** Department of Pediatrics, Shengjing Hospital of China Medical University, Shenyang, China

**Keywords:** autosomal recessive inheritance, Chinese pedigree, febrile convulsions, HSH, novel mutation, TRPM6

## Abstract

**Background:**

Hereditary hypomagnesemia with secondary hypocalcemia (HSH) is a rare autosomal recessive disease due to biallelic TRPM6 mutations. Although the reports of HSH caused by TRPM6 mutations are not very rare, the age of onset in previously reported HSH cases were <1 year.

**Methods:**

We collected and analyzed the clinical data of twin brothers with onset age over 1 year old and performed whole exome sequencing in the patients and their parents. Confirmed by Sanger sequencing, missense mutation was analyzed *in silico*. We also searched Pubmed, and extracted clinical data from case reports and case series with full text in English, reporting original data of patients with TRPM6 mutations.

**Results:**

The twin patients had canonical HSH phenotype with compound novel TRPM6 mutations, p.T87K and c.705dupT, inherited from their father and mother, respectively. T87 is a highly conserved site and T87K is predicted to cause hydrogen bond disruption. We identified 26 articles published between May 28, 2002 to December 31, 2021 which reported a total of 88 patients with TRPM6 mutation. We found that the most common clinical phenotypes were hypomagnesemia, hypocalcemia, and convulsions. However, the age of onset in HSH patients almost always occurred under 12 months old, the twin patients of our study were 18 and 26 months old at onset.

**Conclusion:**

We identified two novel TRPM6 mutations in a Chinses family with HSH, and showed that the age of onset with c.704c-c.705(exon7)insT and c.260(exon4)C>A mutation in TRPM6 was much later than other mutations and would be much less serious.

## Introduction

Hypomagnesemia can lead to convulsions. Magnesium is mainly associated with neuromuscular conduction and cardiovascular tone, and its deficiency can reduce the release of acetylcholine in neuromuscular junctions and nerve terminals, playing an important part in antagonizing peripheral nerve function. Symptoms, de Boer et al. (1) like increasing nerve excitability similar to that associated with hypocalcemia, appear when magnesium is deficient in the body. When hypomagnesemia accompanies hypocalcemia, the symptoms of convulsions are more severe.

Hereditary hypomagnesemia with secondary hypocalcemia (HSH, MIM: #602014) is a rare autosomal recessive disease due to homozygous or compound heterozygous mutations in the TRPM6 gene. HSH as a disorder of significantly decreased serum magnesium levels, usually <0.7 mmol/L, accompanied by secondary hypocalcemia. In patients with HSH, muscle weakness, tetany that does not respond well to antispasmodic medications, and polyuria are common in their infantile period (2). HSH needs to be differentially diagnosed to distinguish it from epilepsy. Currently, approximately 100 cases are documented (ORPHA:30924, Ophanet), and no dominant inherited or incomplete penetrance was observed in affected families (11 male and 2 female patients from 3 inbred families) (2, 3).

TRPM6 was the common pathogenic genes for HSH. Recent reports indicate that HSH in a Chinese family was mainly caused by TRPM6 mutations (4). TRPM6 gene expression is located on chromosome 9q22, together with TRPM7, which encodes a calcium and magnesium permeable divalent cation channel, TRPM6 assembles a homo or heterotetramer to form the functional unit (5). It plays an important role in maintaining serum magnesium homeostasis. When this gene is incorrectly encoded, intestinal absorption and magnesium absorption from the renal tubules fail, and serum magnesium reduces, a block in parathyroid hormone release from the parathyroid gland in severe hypomagnesemia, Quitterer et al. (6) resulting in secondary hypocalcemia. Although the reports of HSH caused by TRPM6 mutations are not very rare, the age of onset in Walder et al. (7) previously reported HSH cases were <1 year. Here, we report an HSH family where the age of onset in a pair of twin brothers was older than 1 year, as well as two new TRPM6 mutations that have not been previously reported.

## Materials and Methods

### Clinical Descriptions

#### Patient I

Patient I was the younger of the fraternal twin brothers born *via* C-section at week 36 of pregnancy. His developmental history was normal. His mother and the maternal grandfather had histories of childhood febrile convulsions. The other members of his family did not experience similar clinical manifestations. Between the 1.5–2 years age, the boy frequently experienced tetany. At 25 months old, he was brought to our hospital for convulsions with fever (temperature 39.5°C). His typical symptoms or signs, lasting for 2–3 min, manifested as loss of consciousness with tonoclonic spasm, strabismus and cyanotic lips. The patient had another similar convulsions with fever (temperature 38.5°C) the next day. An emergency blood test during the second convulsions showed that magnesium was 0.25 mmol/L (normal magnesium is 0.67–1.15 mmol/L), and calcium was 1.98 mmol/l (normal calcium is 1.9–2.6 mmol/L).

The patient was then treated with MgSO4 *via* an intravenous supplement. After treatment, his magnesium and calcium levels elevated to 0.57 and 2.43 mmol/L, respectively. Six months later, the convulsions recurred with decreased levels of magnesium (0.23 mmol/L) and calcium (1.87 mmol/L) and his symptoms and ion levels were restored following a higher dose of MgSO4 by intravenous supplement. His convulsions were now well-controlled by individual oral potassium magnesium aspartate (PMA) therapy for 1 year.

#### Patient II

Patient II is the fraternal twin brother of patient I. His development history was also normal. At 26 months old, patient II had febrile convulsions, the manifestations being broadly similar to those of his identical twin, lasting for 1 min. The serum tests at that time showed that magnesium was 0.29 mmol/L, and calcium was 1.86 mmol/L. He also underwent oral PMA therapy without a guidance under the professional physician and gene analysis. Approximately 1 month later, without regular reexamination, febrile convulsions occurred again. His serum magnesium was 0.28 mmol/L and calcium was 2.04 mmol/L. After intravenous magnesium supplement administration separately, his magnesium elevated to 0.64 mmol/L and calcium to 2.54 mmol/L. Then he had a fever again because of the infection of Rotavirus. This time, the convulsions didn't appear, and other symptoms were completely controlled.

The two children's neurological examinations were negative. The blood regular tests, serum etiology detection, blood bacterial culture and video electroencephalogram examinations all ruled out a central nervous system infection. To exclude out metabolic illnesses, blood ammonia, lactic acid, thyroid function, parathyroid gland function, blood glucose, blood and urine genetic metabolism were all evaluated. Cerebral magnetic resonance imaging (MRI) showed the Patient I had a big pillow pool, and the Patient II was normal, which ruled out intracranial mass lesions and cerebrovascular disease.

The treatment of these twins relied on oral and intravenous magnesium supplementation. Reexaminations were done every other day. Patient I was treated by individual treatment of intravenous magnesium dose (MgSO4^*^7H2O 400mg+40mL5%Glucose) which the magnesium ion(Mg2+) was 1.03 mmol/kg per day, once a day, seven days in a row. The level of blood magnesium elevated to around 0.6mmol/L, the treatment was changed to individual oral magnesium at 0.62 mmol/kg per day. The treatment for Patient II was just like his cousin, he received the same dose of intravenous magnesium ion. After 4 days of individual intravenous magnesium, the level of magnesium elevated to 0.64mmol/L. Then he also changed to individual oral magnesium at 0.62 mmol/kg per day.

### Mutation Analysis

We collected 2 ml peripheral blood samples from the patients and their parents for whole-exome sequencing (WES). Sanger sequencing was applied to confirm the candidate variant in both patients and their parents.

The WES was performed by Chigene Corp. (Beijing, China). Genome DNA was extracted, purified and quality control (QC) validated, followed by whole exome library construction using xGen Exome Research Panel v1.0 (IDT, Iowa, USA). High-throughput sequencing was performed on a NovaSeq 6000 sequencer (Illumina, USA), as well as raw data cleaning and QC validation following the instructions of the manufacturer. Paired-end reads were aligned to the ensemble reference genome, GRCh37/hg19 using the Burrows-Wheeler Aligner. Single nucleotide polymorphisms (SNP) and short insertions and deletions (<50 bp) calling was conducted using the Genome Analysis Toolkit software package. GnomAD, ESP, 1,000 Genome Project, dbSNP, and Chigene (in-house) databases were used for Mutation Annotation Format annotation; Provean, Sift, Polypen2, Mutationtaster, M-Cap, and Revel softwarepackages were used for variant pathogenicity prediction; MaxEntScan, dbscSNV and GTAG software packages were used to predict splice alteration; ClinVar, Online Mendelian Inheritance in Man, and Human Gene Mutation Database pro databases were used for disease annotation. The pathogenicity of variants was validated according to the American College of Medical Genetics (ACMG) clinical practice guidelines. We used UCSF Chimera for the mac OS version 1.15 to evaluate missense mutation in TRPM6 with the 3-dimensional model (id: Q9BX84, AlphaFold, https://alphafold.ebi.ac.uk/) (7). Further *in silico* analysis of the mutation was performed using VarMap, an online tool for protein structural annotations (https://www.ebi.ac.uk/thornton-srv/databases/cgi-bin/VarSite/GetPage.pl?uniprot_acc=n/a&template=VarMap.html). NCBI BLASTP and Multiple Alignment Viewer were used to compare the sequence conservation.

### Review of Patients Reported in Literature and Statistical Analysis

To delineate the clinical characteristics associated with TRPM6 mutations, we conducted a systematic search of the medical literature on TRPM6 mutations using the PubMed database from May 28, 2002 to December 31, 2021 for related published articles. The types of literature included isolated case reports and case series. We used search terms, “TRPM6” and “Hypomagnesemia with secondary hypocalcemia,” “HSH,” “convulsion,” “seizure,” or “tetany.” Full-text articles were obtained from journals' websites. We analyzed age of onset, gender, clinical symptoms, laboratory findings, type of mutation, and nucleotide change of patients with TRPM6 gene variant. The clinical manifestations and genetic results of all cases reported were analyzed and summarized by using SPSS version 26.0 (IBM, NY).

## Result

**Figure 1:**
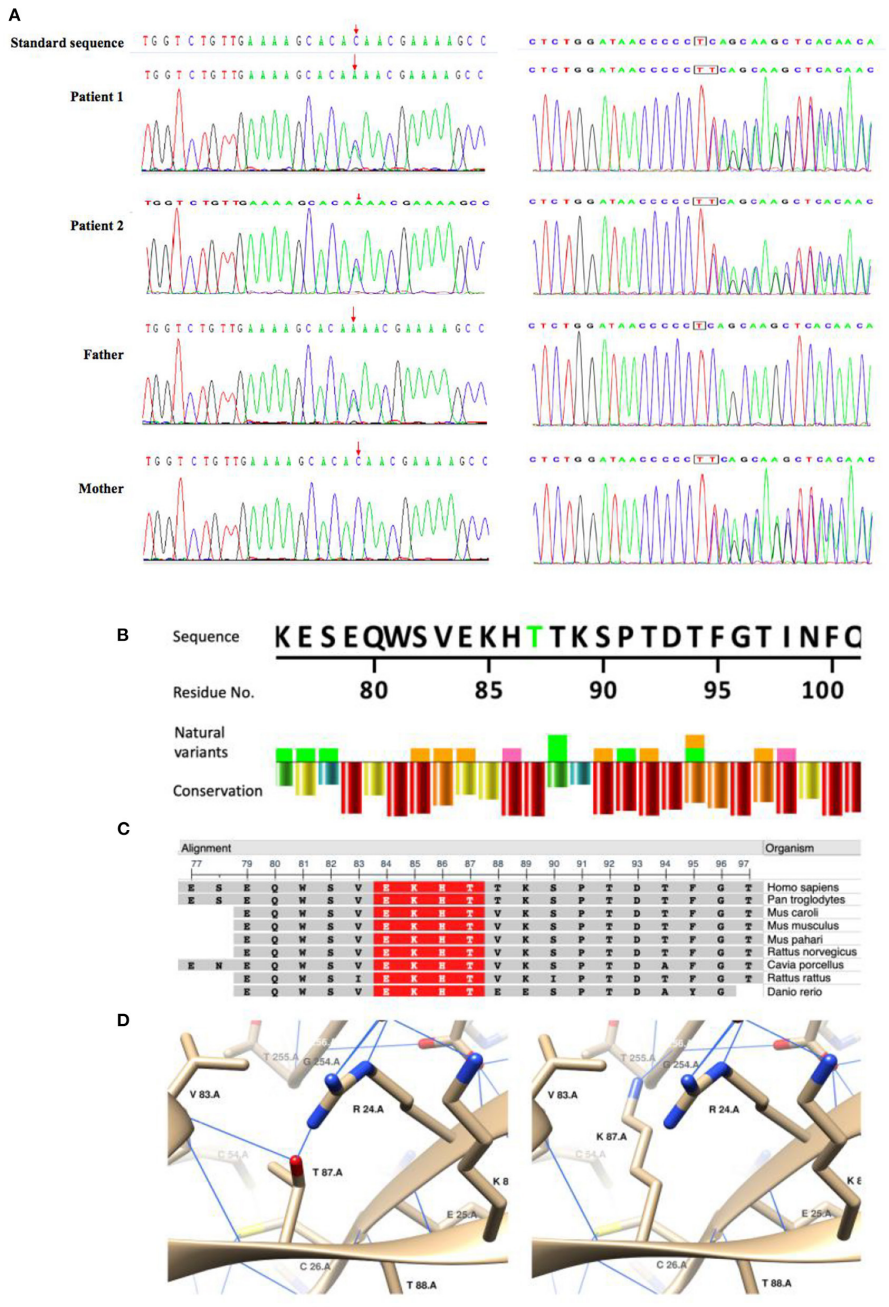
Sanger sequencing of the compound heterozygous mutations and *in silico* analysis of the missense variant(T87K)in TRPM6. **(A)** Sanger sequencing confirmed compound heterozygous mutations in the patients and their parents. **(B–D)** *In silico* analysis of TRPM6T87K. A natural variant and conservation of TRPM6T87. Residue conservation from lowest (purple) to highest (red). Highly conservative sequence (red) in multiple organisms. Generated using NCBI Multiple Sequence Alignment Viewer, Version 1.20.1. C3D structuring of T87 and K87. Blue lines show the hydrogen bonds.

We identified compound heterozygous variants NM_017662.4: c.260C>A/p. in T87K and c.705dupT, in TRPM6 in both patients (Figure 1A). T87K is a variant of uncertain significance (VUS) according to the ACMG guidelines (PM2 + PM3 + PP3), and frameshift mutation c.705dupT is likely pathogenic (PVS1 + PM2).

An *in silico* study showed that T87K is potentially pathogenic (https://www.ebi.ac.uk/thornton-srv/databases/cgi-bin/VarSite/GetPage.pl?uniprot_acc=Q9BX84&template=resreport.html&res=T87K): T87K is a highly conservative site (Figures 1B,C); which makes a hydrophobic to hydrophilic change of the residue and K87 was predicted to generate hydrogen bonds (H-bond) with G254 and to interrupt H-bonds with R24 and V83(Figure 1D), which indicated a potential change of the tertiary structure.

To describe the phenotype and mutation spectrum of the TRPM6 mutations, we collected the clinical information of 88 patients, as well as more detailed information (Supplementary Table 1) that we needed, from 26 articles involving 67 families (2, 4, 8–32). We confirmed that the mutation locations of these twins had not been previously reported. Through summarizing and analyzing the information of these patients, we found that 92.05% (81/88) of patients had the classical clinical manifestations of HSH. Among the patients with HSH, the age of onset ranged from 2 days to 12 months, most patients exhibited symptoms before 3 months, it was more common in females (43/81), with a sex ratio of 1.13:1. All patients with HSH had convulsions; 19.75% (16/81) of patients associated diarrhea; 7.00% (7/10) of patients had various nervous system symptoms, including three with intellectual disability and three with psychomotor retardation; one had dwarfism; one had dysmporphic facial features and microcephaly; one had cardiac arrhythmia; one had hyperactivity; and one had paranoid delusions; three had a history of febrile convulsions. Among 81 symptomatic patients, the initial serum magnesium concentration ranged between 0.05 and 0.53 mmol/L, with an average of 0.25 ± 0.17 mmol/L. The initial serum calcium concentration was 0.12–2.6 mmol/L, with an average of 1.70 ± 0.47 mmol/L. After regular magnesium replacement therapy, most patients showed an elevation of serum magnesium. The mutant homozygote was found in 59.52% (50/84) patients including four types of genetic mutation. The relationship among the four types of mutations, and symptoms are shown in Table 1. Most of mutations were splice site (27/88), followed by missense (26/88) and frame shift (25/88), the least was nonsense (23/88).

**Table 1:** Detailed clinical phenotypes of patients with TRPM6 mutations.

**Type of Mutation**	**Number of patients with HSH**	**Age of onset**	**Convulsion**	**Diarrhea**	**Psychomotor retardation**	**Intellectual disability**
Non-sense	23/88	2d-5m (mean 7.2w)	13	3	1	1
Missense	26/88	1w-9m (mean 12.3w)	13	1	0	0
Frame shift	25/88	1w-9m (mean 10.3w)	16	5	2	1
Splice site	27/88	2d-1y (mean 9.1w)	18	3	1	2
**Type of Mutation**	**Hyperactivity**	**Paranoid delusions**	**Facial deformity**	**Microcephaly**	**Dwarf**	**Cardiac arrhythmia**	**Asymptomatic**
Non-sense	0	-	1	1	1	1	2
Missense	0	-	0	0	0	0	1
Frame shift	1	-	0	0	0	0	3
Splice site	0	-	2	1	0	0	2

The patients were dependent on oral and intravenous magnesium supplementation. Parenteral magnesium supplementation was clearly reported in 32 cases, subcutaneous in 8 cases, unstated in 1 case, and intravenous in 23 cases. The 9 patients in the report confirmed the measurement of intravenous magnesium supplementation, with average = 1.567mmol/kg^*^d, measure of quartile method is [0.508 (4.765, 0.365)] mmol/kg^*^d. the maximum 8.23 mmol/kg^*^d and the minimum 0.20 mmol/kg^*^d. Clinical symptoms disappeared, blood magnesium and calcium level increased were the indications for most of the patients. And the average dose of oral magnesium is 1.91 mmol/kg^*^d, the minimum is 0.20 mmol/kg^*^d, the maximum is 20.57 mmol/kg^*^d.

**Figure 2:**
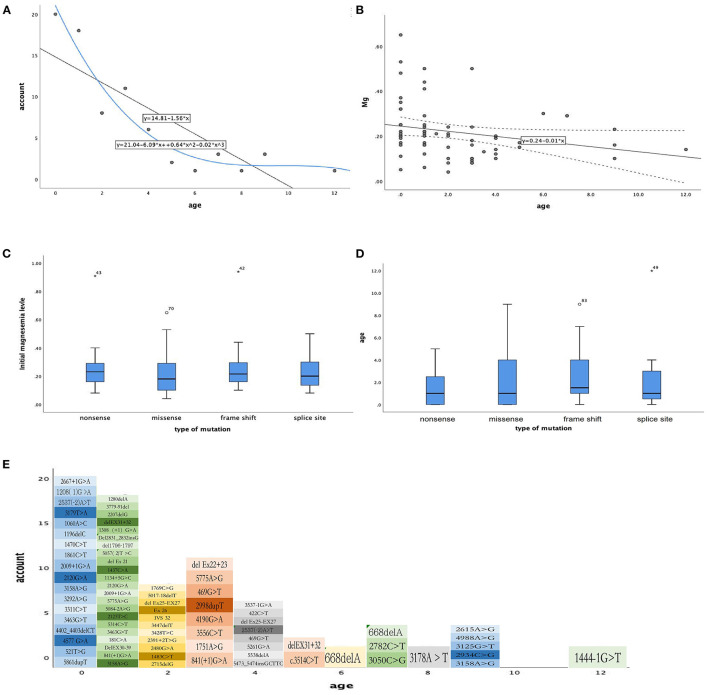
The relationship between phenotype and phenotype / genotype in patients with HSH induced by TRPM6. **(A)** The age of month (X-axis), the number of every month of age (Y-axis), Spearman correlation analysis, r = −0.855, *p* = 0.001 < 0.05. **(B)** The age of month (X-axis), initial magnesium level (mmol/L, Y-axis), Spearman correlation analysis, r = −0.342, *p* = 0.005 < 0.05. **(C)** Types of mutation (X-axis), initial magnesium level (mmol/L, Y-axis), Kruskal–Wallis, H-test *p* = 0.545 > 0.05. **(D)** Types of mutation (X-axis), age of onset (Y-axis), Kruskal–Wallis, H-test, *p* = 0.848 > 0.05. **(E)** The mutation site for age of onset.

**Figure 3:**
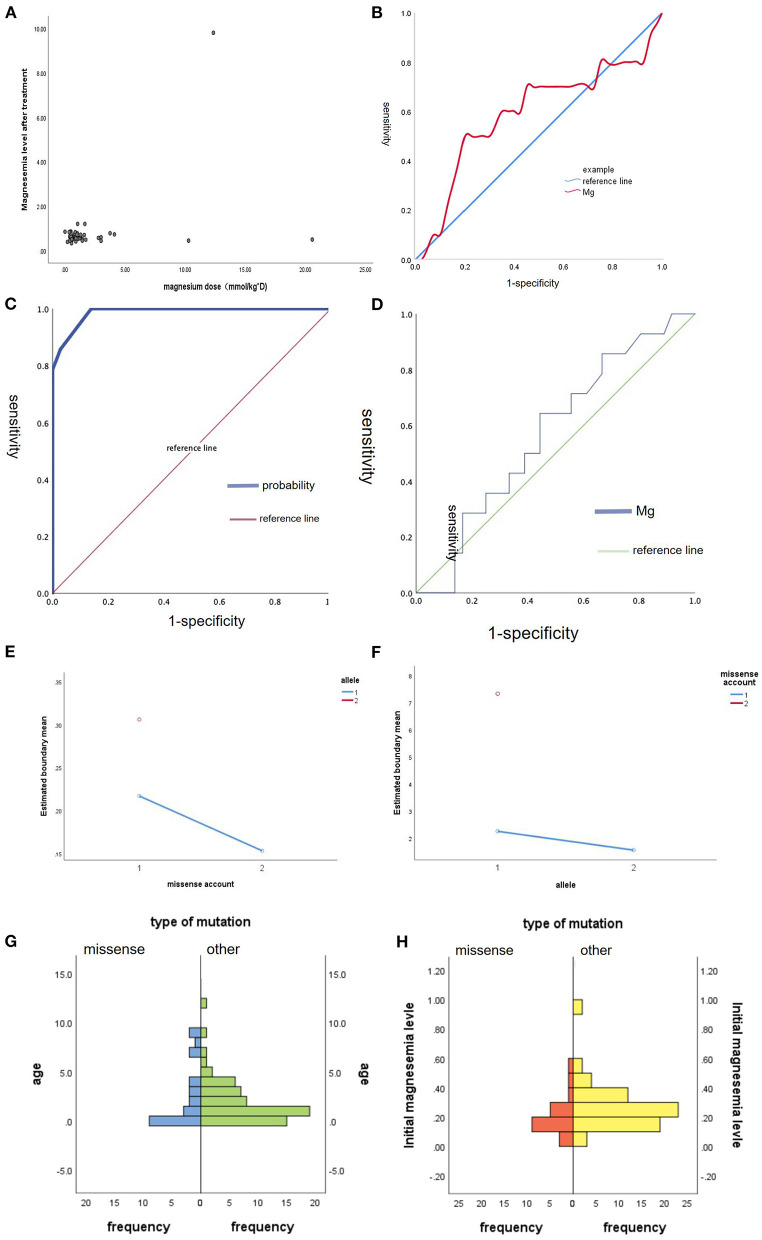
The maintenance treatment in patients with HSH induced by TRPM6. **(A)** The scatter plot between the dose of oral magnesium (mmol/kg*d, X-axis) and the level of magnesium after treatment (mmol/L, Y-axis). **(B)** ROC curve between the dose of magnesium and whether the level of magnesium reach 0.5 mmol/L, AUC = 0.594 > 0.5. **(C)** ROC curve between the prediction probability (dose and serum magnesium) and the diarrhea, AUC = 0.987 > 0.5. **(D)** ROC curve between the dose of magnesium and the occurrence of diarrhea, AUC = 0.570 > 0.5. **(E)** Main effect graph between the combination effect of number of missense and non-allelic mutation on the initial magnesium level. **(F)** Main effect graph between the combination effect of one missense mutation and whether an allele mutation on the age of onset. **(G)** The difference between missense and other type of mutation on the age of onset, Mann-Whitney U-test, *p* = 0.939 > 0.05. **(H)** The difference between missense and other type of mutation on the initial magnesium level, Mann-Whitney U-test, *p* = 0.052 > 0.05.

And we did some statistics amone the age of onset, initial magnesium level and the type of mutation (Figure 2). We also count the dose of magnesium (Figure 3). The Spearman correlation analysis showed there was a closely correlation between the age of onset and the account of the patients (Figure 2A), meanwhile, the age of onset was relevant to the initial magnesium level (r = −0.342, *p* = 0.005 < 0.05) (Figure 2B). And the results showed there were no differences among four types of mutation were also analyzed according to the age of onset (Kruskal–Wallis, H-test, *p* = 0.848 > 0.05) (Figure 2C) and initial serum magnesium level (Kruskal–Wallis, H-test, *p* = 0.848 > 0.05) (Figure 2D). And there was no corresponding relation between the dose of oral magnesium and the level of magnesium after treatment through Spearman correlation analysis, *p* = 0.363 > 0.05 (Figure 3A), the scatter plot between the dose of oral magnesium and the level of magnesium after treatment was also showed it (Figure 3B). The high dose of oral magnesium cannot make a corresponding high serum magnesium level. According to the previous literature (33), the magnesium level after treatment was divided into 2 groups (>0.5 mmol/L and ≦0.5 mmol/L). ROC curve was drew between the dose of magnesium and whether the level of magnesium reach 0.5 mmol/L(AUC = 0.594>0.5), through the Youden's index we coule find Cut off value while the maximum dose of magnesium is 1.395 mmol/kg^*^d, when the dose of magnesium higher than 1.395 mmol/kg^*^d, the serum magnesium level cannot ascend correspondingly. As the onset of diarrhea, ROC curve between the prediction probability (dose and serum magnesium) and the diarrhea, AUC = 0.987 > 0.5 (Figure 3C). ROC curve between the dose of magnesium and the occurrence of diarrhea was also made, AUC = 0.570 > 0.5, the Cut off value is 0.875mmol/kg^*^d, the diarrhea may occur when the dose of magnesium over 0.875mmol/kg^*^d (Figure 3D). Blood magnesium concentration after treatment in previous literatures was 0.63 ± 0.17 mmol/L. About 18.37% (9/49) of the patients had complete clinical symptoms control when the blood magnesium concentration was lower than 0.5 mmol/L.

We also did some statistics among the age of onset, initial magnesium level and missense (Figure 3). We did the Main-effect graph to find the influence of allele and count of missense on the age of onset and initial magnesium level (Figures 3E,F). When non-allelic mutation, the more missense, the lower Initial magnesium level, when there is only one missense mutation, the age of onset of allele mutation was earlier than that of non-allelic mutation. And there was no significant difference between missense and other mutation types in initial magnesium ion concentration and age of onset. Mann-Whitney U-test *p* = 0.939 > 0.05. There was no difference between the Missense and other types of mutation on the age of onset. Mann-Whitney U-test *p* = 0.052 > 0.05 (Figures 3G,H). There is no difference between the missense and other types of mutation on the initial magnesium level.

## Discussion

Our twin patients showed canonical HSH: infantile onset hypomagnesemia, hypocalcemia, and convulsions that responded to magnesium intake therapy. As Schlingmann et al. (2) summarized from the study of 28 HSH patients from 21 families, TRPM6 biallelic mutations always cause complete functional loss of TRPM6, which was proven in our twin patients. Severe hypomagnesemia and hypocalcemia may result in permanent central nervous system damage or even death, (2, 4) indicating that early diagnosis and medication is critical to managing HSH. However, in our case, a history of febrile convulsions in the patient and his maternal family members could disrupt the initial diagnosis, which may be explained by the fact that fever intensifies the convulsions caused by hypomagnesemia. The previous literature also showed 3 patient had a history of febrile convulsions, 2 didn't complete the serum examination, 1 occurred after diagnosed (12, 30, 34).

HSH is a rare genetic disease, which is inherited in an autosomal recessive manner. In recent reports, four types of mutation of TRPM6 have been found (8). As Table 1 shows, in these studies, researchers found no correlation between different types of mutations and patients' clinical manifestations (9). Convulsions were the most common symptom.

Magnesium homeostasis depends primarily on the balance between intestinal absorption and renal excretion. Magnesium transport processes in both organ systems along with passive paracellular magnesium flux involve active transcellular magnesium transport consisting of an apical uptake into the epithelial cell and a basolateral extrusion into the interstitium (10). TRPM6 belongs to the transient receptor potential (TRP) superfamily, which consists of various cation permeable channels involved in ion homeostasis and/or signal transduction (11). TRPM6 is closely related to TRPM7, both sharing the unique feature of a serine/threonine kinase domain c-terminally fused to their ion channel domains (35, 36). In contrast to TRPM7, the expression pattern of TRPM6 seems to be more confined, with expression mainly along the gastrointestinal tract and kidney, predominantly in the distal convoluted tubule (DCT), Lomelino-Pinheiro et al. (31) where it is presumably involved in the apical entry of magnesium into epithelial cells (37). The TRPM6 gene mutation could lead to a dysfunction of the gastrointestinal tract and DCT, which would interfere with the apical entry of magnesium into epithelial cells and causes the decline of serum magnesium. According to the analyzed case data in the literature we reviewed, the mean value of blood magnesium is 0.25 ± 0.17 mmol/L, and the mean value of blood calcium is 1.70 ± 0.47 mmol/L. The two cases we report have similar results, with significantly lower values of magnesium than normal and secondary hypocalcemia and convulsions. Consistent with other reports, our two cases had very low magnesium concentrations and secondary hypocalcemia.

The patients in our study had convulsions with fever. We speculate that the convulsions caused by the novel mutation might have been potentially induced or aggravated by fever. As in other metabolic disorders affecting the CNS, a triggering event seems necessary to cause decompensation (38, 39). In the present case, the triggering event might have been a fever episode, which preceded the three acute neurological episodes experienced by the two patients. How a fever leads to decompensation is unknown. However, in patients with compound heterozygous variants, NM_017662.4: c.260C>A/p. in T87K and c.705dupT, in TRPM6, it may be wise to recommend appropriate measures to control the body temperature and avoid catabolic states.

HSH results in electrolyte abnormalities shortly after birth (32). Although the mild hypomagnesemia has been described with transient hypoparathyroidism (40, 41) in the neonatal period. A recurrent hypomagnesemia should be concerning. The age of onset in most HSH patients is under 3 months. The age of onset of the patients in our study was much later than other types and would be much less serious.

In mice, TRPM6 mutations can lead to abnormal development and neural tube defects. The report by Komiya et al. (42), Walder et al. (43) described a Saudi girl with HSH, whose cerebral MRI revealed bilateral ganglia calcification. In our study, the cerebral MR showed the Patient I had a big pillow pool, and the Patient II was normal. Both had a normal developmental history which were consistent with the literature review. We also noticed that 4.5% of patients experienced intellectual disability which might have been caused by the delayed diagnosis and unstable serum magnesium levels (2, 44). The cases of HSH with developmental retardation and cranial imaging changes were rare, a larger sample is needed to confirm the association between this clinical phenomenon and TRPM6 mutation.

As Supplementary Table 1 shows, seven asymptomatic members without classical symptoms of HSH. They all with identified mutation sites exhibited exon mutations, but we found no relation between the exon mutations and their symptoms. We believe that there are some sites in the TRPM6 gene where mutations would result in less serious manifestations than would others, such as in the patients in our study. On comparing the two brothers' symptoms with the 88 patients from the literature, we found that their onset time was later and their developmental progress was not influenced. The same types of mutation may lead to different outcomes. We found that even in the same household, patients with the same mutation, the same family history and the same environment, some would die, while others would suffer symptoms which could be controlled through regular magnesium supplementation. In the families with deaths, the patients' symptoms were more serious than in other families, they would suffer not only convulsions, but also other symptoms, such as intellectual disability, mental retardation, or even malformation.

For the treatment of patients with HSH, intravenous magnesium followed by oral magnesium was the most common, the recommended maintenance dose is 1mmol/kg^*^d orally. However our statistics show the average dose of oral magnesium is 1.91mmol/kg^*^d. Our patients undertook around 0.6mmol/kg^*^d, which is much lower than the recommended dose and the average dose from previous reports. The differences among patients' individual characteristics are huge. The celiac diseases as chronic diarrhea may aggravate the loss of magnesium (45). Therefore, the maintenance dose of oral magnesium should not be too large to prevent the drug-induced diarrhea and supple symptomatic treatment timely.The AUC of ROC curve >0.5 but <0.7 may lead to poor prediction effect, so this range of magnesium is only for reference, every patient with HSH should do regular reexamination of serum magnesium to flexible regulate the dose of magnesium supplement. Some children had complete clinical symptoms control when the blood magnesium concentration was lower than 0.5mmol/L. The vast majority of patients have a good prognosis.

Our statistics cannot find that patients with missense mutations had milder symptoms through age of onset and initial serum magnesium level. While, the main effect graph shows that the number missense and the combination of allele and non-allele may influence the symptoms. The clinical phenotype of the two cases we reported is relatively mild, which may be related to the missense mutations that partially affect protein function. However, due to the rarity of missense, the sample capacity is too small, there may be deviation in the statistical results. An individual with com-heterozygous missense mutation who developed febrile convulsion at the beginning of the disease and was diagnosed after low magnesium was tested (12). Since only one allele E872G mutation in the patient was clearly pathogenic and molecular studies were lacking, the literature only proposed that missense mutation of residual function might be the cause of the undetermined mild phenotype. The parents of the patients reported in the literature were asymptomatic individuals. In our report, there was a history of febrile convulsions in the maternal line (missense carriers) when they were young, but no recurrence without systematic treatment. Therefore, we believe that the residual function of missense can be speculated but cannot be confirmed at present. It is necessary to collect and analyze more samples to confirm this conclusion.

## Conclusion

We present fraternal twin brothers with HSH resulting from compound heterozygous variants, NM_017662.4: c.260C>A/p. in T87K and c.705dupT, in TRPM6. The new TRPM6 mutations appear to be an important cause of HSH which includes the clinical features of febrile convulsions and late onset age. Pediatricians need to consider the diagnosis of HSH in febrile convulsive young children, especially when hypomagnesemia and hypocalcemia are found.

## Data Availability Statement

The datasets for this article are not publicly available due to concerns regarding participant/patient anonymity. Requests to access the datasets should be directed to the corresponding author.

## Ethics Statement

The studies involving human participants were reviewed and approved by Shengjing Hospital of China Medical University. Written informed consent to participate in this study was provided by the participants' legal guardian/next of kin. Written informed consent was obtained from the minor(s)' legal guardian/next of kin for the publication of any potentially identifiable images or data included in this article.

## Author Contributions

YH: conceptualization and writing–review and editing. YZ: data curation and writing–original draft preparation. HW: methodology, software, and validation. LH: project administration, supervision, and funding acquisition. All authors contributed to the article and approved the submitted version.

## Funding

This work was supported by Liaoning Provincial Department of Education Scientific Research Project (QNZR2020012), Henan Neural Development Engineering Research Center for Children Foundation, (SG201905), CAAE Epilepsy Research Fund (CX-B-2021-02).

## Conflict of Interest

The authors declare that the research was conducted in the absence of any commercial or financial relationships that could be construed as a potential conflict of interest.

## Publisher's Note

All claims expressed in this article are solely those of the authors and do not necessarily represent those of their affiliated organizations, or those of the publisher, the editors and the reviewers. Any product that may be evaluated in this article, or claim that may be made by its manufacturer, is not guaranteed or endorsed by the publisher.
