# Supplementary material for: Case Report: Novel TRPM6 Mutations Cause Hereditary Hypomagnesemia With Secondary Hypocalcemia in a Chinese Family and a Literature Review
Source: Front Pediatr. 2022 Jul 12;10:912524. doi: 10.3389/fped.2022.912524 (PMC9315244; doi:10.3389/fped.2022.912524)
Supplement: Supplementary file 1 [file Table_1.doc]

Suppl. Table 1: Transient Receptor Potential Mealstatin‑6 (TRPM6) Channel defects presenting as hypomagnesemia with secondary hypocalcemia - Review of case reports and case series

| Ref. | No. of the patients | Age of Onset | Gender | Clinical  Symptom | Initial Mg^2+^  (mmol/L) | Mg^2+^after treatment  (mmol/L) | Initial  Ca^2+^  (mmol/L) | Ca^2+^ after treatment  (mmol/L) | source | type of mutation | nucleotide change | type of nucleotide change | Exons | family history |
| --- | --- | --- | --- | --- | --- | --- | --- | --- | --- | --- | --- | --- | --- | --- |
| Schlingmann KP, et al. 2002^[16]^ | p1 | 9w | F | convulsion | 0.21 | 0.59 | 1.63 | N/A | Ho | nonsense | 1769C>G | substitution | Exon 16 | + |
|  | p2 | 3w | M | intellectual disability, convulsion | N/A | 0.57 | 1.29 | N/A | Ho | splice site | 2667+1G>A | substitution | IVS 20 | + |
|  | p3 | 4m | F | diarrhea, convulsion | 0.1 | 0.55 | 2.5 | N/A | CHe | splice site+  missense | [3537-1G>A]+[422C>T] | substitution  + substitution | IVS 25+ Exon 4 | - |
|  | p4 | 5w | M | diarrhea, convulsion | 0.41 | N/A | 1.88 | N/A | CHe | frame shift +frame shift | [1280delA]+[3779-91del] | deletion  + deletion | Exon 11+ Exon 26 | - |
|  | p5 | 5w | F | convulsion | 0.17 | 0.55 | 1.5 | N/A | Ho | frame shift | 2207delG | deletion | Exon 17 | + |
|  | p6 | 5w | F | convulsion | 0.22 | 0.55 | 1.6 | N/A | Ho | frame shift | 2207delG | deletion | Exon 17 | + |
| Schlingmann KP,et al, 2005^[4]^ | p1.1 | 5m | M | convulsion | 0.15 | 0.86 | 1.94 | N/A | Ho | nonsense | delEX31+32 | deletion | Exon 31+32 | + |
|  | p1.2 | 5w | M | cardiac arrhythmia, convulsion | 0.22 | 0.85 | 1.73 | N/A | Ho | nonsense | delEX31+32 | deletion | Exon 31+32 | + |
|  | p2 | 6w | M | diarrhea, convulsion | 0.21 | 0.6 | 1.63 | N/A | Ho | splice site | 1308（+1）G>A | substitution | IVS 11 | + |
|  | p3 | 3m | F | convulsion | N/A | 0.6 | 1.74 | N/A | Ho | frame shift | del Ex22+23 | deletion | Exon 22+23 | + |
|  | p4 | 2m | F | convulsion, diarrhea | 0.2 | 0.56 | 1.31 | N/A | He | frame shift +? | [5017-18delT]+? | deletion | Exon 30+? | - |
|  | p5 | 6w | F | diarrhea, convulsion | N/A | 0.51 | N/A | N/A | Ho | frame shift | Del2831_2832insG | translocation | Exon 21 | + |
|  | p6.1 | 4m | M | convulsion, diarrhea | 0.19 | N/A | N/A | N/A | Ho | frame shift | del Ex25-EX27 | deletion | Exon 25- 27 | + |
|  | p6.2 | 2m | F | convulsion, diarrhea | 0.1 | 0.44 | 1.66 | N/A | Ho | frame shift | del Ex25-EX27 | deletion | Exon 25-7 | + |
|  | p7.1 | 3m | M | convulsion | 0.09 | 0.33 | 1.6 | N/A | Ho | splice site | 5775A>G | substitution | Exon 36 | + |
|  | p7.2 | N/A | M | - | 0.16 | 0.53 | 1.75 | N/A | Ho | splice site | 5775A>G | substitution | Exon 36 | + |
|  | p8.1 | 6m | F | convulsion, hyperactivity | 0.3 | 0.61 | 1.75 | N/A | He | frame shift +? | 668delA +? | deletion +? | Exon 6 +? | - |
|  | p8.2 | N/A | F | - | N/A | N/A | N/A | N/A | He | frame shift +? | 668delA +? | deletion +? | Exon 6 +? | - |
|  | p8.3 | 7m | M | - | N/A | N/A | N/A | N/A | He | frame shift +? | 668delA +? | deletion +? | Exon 6 +? | - |
|  | p9 | 3w | F | diarrhea, convulsion | 0.2 | 0.78 | 1.35 | N/A | He | splice site | 1208(1)G>A + ? | substitution+? | N/A | - |
|  | p10 | 7m | M | diarrhea, convulsion | 0.29 | 0.5 | 1.6 | N/A | He | missense+? | 2782C>T+? | substitution+? | Exon 21+? | - |
|  | p11 | 5w | F | diarrhea, convulsion | 0.29 | 0.49 | 1.45 | N/A | CHe | frame shift +nonsense | [del1796_1797] +[5057(2)T>C ] | deletion  + substitution | Exon 16 + IVS 30 | - |
|  | p12.1 | 2w | F | intellectual disability, diarrhea, convulsion | 0.37 | 0.58 | N/A | N/A | He | splice site | 2537(-2)A>T + ? | substitution+? | IVS 19 +? | - |
|  | p12.2 | 4m | F | convulsion | N/A | N/A | N/A | N/A | He | splice site | 2537(-2)A>T + ? | substitution+? | IVS 19+? | - |
|  | p13 | 4w | F | convulsion | 0.44 | 0.45 | 1.7 | N/A | Ho | frame shift | del Ex 21 | deletion | Exon 21 | + |
|  | p14 | 3m | M | convulsion | 0.1 | 0.5 | 1.45 | N/A | Ho | missense | 469G>T | substitution | Exon 5 | + |
|  | p15 | 2m | F | convulsion | N/A | 0.62 | N/A | N/A | CHe | missense + splice site | Ex 26 +IVS 32 | N/A | Exon 26 + IVS 32 | - |
|  | p16 | 3w | F | convulsion | 0.2 | 0.52 | 1.72 | N/A | Ho | splice site | 2667 +1G>A | substitution | IVS 20 | + |
| Habeb AM et al, 2012^[31]^ | p1 | 3m | F | convulsion | 0.24 | 0.59 | 1.6 | 2.43 | Ho | frame shift | 2998dupT | insertion | N/A | + |
| Altıncık A et al, 2016^[32]^ | p1 | 2m | F | diarrhea, convulsion | <0.15 | 0.5-0.58 | 2.48 | 2.22-2.29 | Ho | frame shift | 3447delT | deletion | N/A | - |
| Katayama K et al, 2015^[14]^ | p1 | 3m | F | diarrhea, dwarf, convulsion | 0.1 | 0.53-0.74 | 1.55 | N/A | Ho | nonsense | 4190G>A | substitution | N/A | - |
| Esteban-Oliva D et al, 2009^[46]^ | p2 | 16d | F | convulsion | 0.32 | 0.45 | 1.78 | 2.19 | Ho | nonsense | 1470C>T | substitution | Exon 12 | - |
| Reetta Jalkanen  et al, 2006^[33]^ | p1 | N/A | F | - | 0.4 | 0.73 | 2.06 | 2.54 | CHe | nonsense + splice site | 1437C>A+1134+5G>C | substitution  + substitution | Exon 12 | + |
|  | p2 | 1m | f | convulsion | 0.24 | N/A | 1.8 | N A | CHe | nonsense + splice site | 1437C>A+1134+5G>C | substitution  + substitution | Intro9 | + |
|  | p3 | 2w | F | convulsion | 0.17 | 0.52-0.88 | 1.19 | 2.32-2.73 | CHe | missense | 1060A>C+521T4G | substitution+substitution | Exon 9 | - |
|  | p4 | 2w | F | convulsion | 0.19 | 0.45-0.82 | 1.4 | 2.2-2.47 | CHe | missense | 1060A>C+521T4G | substitution  + substitution | Exon 5 | - |
|  | p5 | 1m | F | facial deformity, microcephaly, psychomot retardation, convulsion | 0.16 | 0.29-0.84 | 1.54 | 1.83-2.7 | CHe | splice site + nonsense | 2009+1G>A+2120G>A | substitution  + substitution | Intro16 | - |
|  | p6 | 2D | F | convulsion | 0.25 | N/A | 1.65 | N/A | CHe | splice site + nonsense/X-chromosome translocation | 2009+1G>A+2120G>A | substitution  + substitution | Exon 17 | N/A |
| Zhen Zhao et al, 2013^[5]^ | p1.1 | 3w | F | convulsion | 0.22 | N/A | 2.02 | N/A | CHe | frame shift + nonsense | 1196delC +4577 G>A | deletion +substitution | Exon10 and 26 | + |
|  | p1.2 | 2w | F | convulsion, intellectual disability | 0.32 | N/A | 2.22 | N/A | CHe | frame shift + nonsense | 1196delC +4577 G>A | deletion +substitution | Exon10 and 26 | + |
|  | p2 | 3w | M | convulsion, paranoid delusions | N/A | N/A | N/A | N/A | N/A | N/A | N/A | N/A | N/A | + |
|  | p3 | N/A | F | - | 0.94 | N/A | 2.35 | N/A | He | frame shift | 1196delC | deletion | Exon10 | - |
|  | p4 | N/A | M | - | 0.91 | N/A | 2.28 | N/A | He | nonsense | 4577 G>A | substitution | Exon26 | - |
| Tulay Guran et al, 2012^[34]^ | p1.1 | 3m | M | convulsion | 0.16 | 0.39 | 1.8 | 2.7 | Ho | nonsense | c.3556C>T | substitution | N/A | - |
|  | p1.2 | 3m | F | convulsion | 0.08 | 0.45 | 1.8 | 2.1 | Ho | nonsense | c.3556C>T | substitution | N/A | - |
|  | p2 | 1m | M | convulsion | 0.2 | 0.41 | 2.4 | 2.6 | Ho | splice site | 5775A>G | substitution | N/A | - |
|  | p3 | 1y | M | convulsion | 0.14 | 0.75 | 2.6 | 2.6 | He | splice site | 1444-1G>T | substitution | N/A | + |
|  | p4.1 | 1m | M | convulsion | 0.5 | 0.58 | 1.8 | 2.4 | Ho | splice site | 5775A>G | substitution | N/A | - |
|  | p4.2 | 3m | F | convulsion | 0.5 | 0.66 | 1.7 | 2.3 | Ho | splice site | 5775A>G | substitution | N/A | - |
|  | p5 | 3m | F | convulsion | 0.08 | 0.66 | 2.1 | 2.5 | N/A | N/A | N/A | N/A | N/A | - |
| Roxanne Y. Walder et al.2002^[35]^ | p1 | N/A | N/A | convulsion | N/A | N/A | N/A | N/A | Ho | splice site | 2009+1G>A | substitution | Intro16 | N/A |
|  | p2 | N/A | N/A | convulsion | N/A | N/A | N/A | N/A | Ho | splice site | 2009+1G>A | substitution | Intro16 | N/A |
|  | p3 | N/A | N/A | convulsion | N/A | N/A | N/A | N/A | Ho | splice site | 2009+1G>A | substitution | Intro16 | N/A |
|  | p7 | N/A | N/A | convulsion | N/A | N/A | N/A | N/A | Ho | nonsense | 1420C>T | substitution | Exon12 | N/A |
|  | p8 | N/A | N/A | convulsion | N/A | N/A | N/A | N/A | He | nonsense | 166C>T | substitution | Exon 4 | N/A |
|  | p9 | N/A | N/A | convulsion | N/A | N/A | N/A | N/A | Ho | splice site | 1010+5G>C transversion | substitution | Intron 8 | N/A |
|  | p10 | N/A | N/A | convulsion | N/A | N/A | N/A | N/A | Ho | splice site | 3209–68A>G transition | substitution | Exon 24 | N/A |
| Vladimir Chubanov  et al, 2007^[36]^ | p1 | 7m | N/A | convulsion | 0.29 | 0.93 | N/A | N/A | He | missense | 3050C>G | substitution | Exon 11 | - |
| Sergio Lainez  et al, 2014^[30]^ | p1 | 2m | M | convulsion | 0.08 | 0.69 | 1.94 | 2.42 | CHe | splice site | [3428T>C]+[2391+2T>G] | substitution  +substitution | N/A | - |
|  | p2 | infancy | F | convulsion | 0.05 | 0.5 | 1.78 | 2.4 | Ho | missense | 3158A>G | substitution | N/A | + |
|  | p3 | 4m | F | convulsion | 0.2 | 0.53 | 1.6 | 2.8 | CHe | missense + missense | [469G>T]+[5261G>A] | substitution  +substitution | N/A | - |
|  | p4 | 6w | F | convulsion | 0.12 | 0.82 | 1.6 | N/A | CHe | Missense + splice site | [5084-2A>G]+[2123T>C] | substitution  +substitution | N/A | - |
|  | p5 | 9m | F | convulsion | 0.1 | 0.67 | 1.47 | 2.67 | CHe | Missense + missense | [2615A>G]+[4988A>G] | substitution  +substitution | N/A | - |
| Masaki Shimizu  et al, 2014^[37]^ | p1 | 1m | M | convulsion | 0.06 | N/A | 1.25 | N/A | N/A | missense | 5314C>T | substitution | N/A | N/A |
| Michelle Coulter  et al, 2015^[38]^ | p1 | 8d | M | convulsion | 0.21 | 0.69 | 1.78 | N/A | CHe | frame shift + missense | 5861dupT+1861C>T | insertion  + substitution | N/A | - |
| Marianne C Astor  et al, 2015^[13]^ | p1 | 9m | F | diarrhea, convulsion | 0.16 | 0.5-0.6 | 1.3 | N/A | CHe | missense + missense | 2934C>G+3125G>T | substitution  +substitution | Exon 22+ Exon 23 | - |
|  | p2 | 3w | M | diarrhea, convulsion | 0.25 | 0.6 | 1.9 | N/A | Ho | nonsense | 3463G>T | substitution | Exon 25 | - |
|  | p3.1 | 1m | M | diarrhea, convulsion | N/A | 0.6-0.7 | N/A | N/A | Ho | nonsense | 3463G>T | substitution | Exon 25 | + |
|  | p3.2 | 10d | M | convulsion | N/A | 0.7-0.8 | 3.9 | N/A | Ho | nonsense | c.3463G>T | substitution | Exon 25 | + |
|  | p4 | 1m | M | convulsion | 0.25 | 0.7 | 1.35 | 0.58 | CHe | missense + nonsense | 181C>A+Del | substitution  + deletion | Exon4 + Exon30-39 | - |
| Kekatpure MV et al, 2016^[39]^ | p1 | 2m | N/A | convulsion | 0.04 | 0.58-0.70 | 1.5 | N/A | N/A | missense | 2480G>A | substitution | IVS18 | - |
| Peng J  et al, 2019^[40]^ | p1 | N/A | N/A | convulsion | N/A | N/A | N/A | N/A | He | missense | 342_353delTTCTTATGATAC | deletion | N/A | N/A |
| M. Kamran Azim  et al, 2018^[41]^ | p1 | 15d | M | convulsion | 0.16 | 0.41 | 1.65 | 3.78 | Ho | frame shift | 4402_4403delCT | deletion | Exon 26 | + |
| Sare Gülfem Özlü et al, 2019^[33]^ | p1 | 8m | M | convulsion | N/A | N/A | N/A | N/A | Ho | missense | 3178A > T | substitution | N/A | - |
| Takeshi Goda et al,2019^[42]^ | p1 | 2m | M | convulsion | 0.24 | 1.1-1.3 | 0.78 | N/A | CHe | Nonsense + nonsense | 2715delG+1483C>T | deletion+ substitution | Exon13+Exon21 | - |
| Yang Zhigang et al, 2019^[44]^ | p1 | 4m | F | convulsion | 0.14 | N/A | 1.53 | N/A | Ho | frame shift | 5538delA | deletion | N/A | - |
| Jamie Willows et al, 2019^[45]^ | p1.1 | 20d | F | convulsion | 0.35 | 0.4-0.6 | N/A | N/A | Ho | missense | c.3292A>G | substitution | N/A | + |
|  | p1.2 | 18d | F | convulsion | 0.53 | 0.7–0.9 | normal | N/A | Ho | missense | c.3292A>G | substitution | N/A | + |
|  | p1.3 | 7d | M | - | 0.6-0.7 | 0.7–1.0 | normal | N/A | Ho | missense | c.3292A>G | substitution | N/A | + |
| Tan Jianqiang,et al, 2019^[47]^ | p1 | <1w | M | convulsion | 0.11 | N/A | 1.38 | N/A | Ho | missense | c.3311C>T | substitution | N/A | + |
| Yücel Hüsniye, et al, 2021^[12]^ | p1 | 4m | M | convulsion | 0.12 | 0.78 | 0.12 | N/A | Ho | frameshift | c.5473_5474insGCTTC | insertion | N/A | - |
| Lomelino-Pinheiro Sara,et al, 2020^[48]^ | p1 | 27d | M | convulsion | 0.48 | 0.64-1.02 | 1.3 | N/A | Ho | missense | c.3179T>A | substitution | N/A | - |
| Bayramoğlu Elvan, et al, 2021^[49]^ | p1 | 3m | F | convulsion | 0.18 | 0.71 | 1.62 | 2.52 | Ho | missense | c.1751A>G | substitution | Exon 16 | - |
|  | p2 | 1.5m | M | convulsion | 0.21 | 0.51 | 1.92 | 2.37 | Ho | splice site | c.841(+1)G>A | substitution | IVS7 | - |
|  | P3 | 3.5m | M | convulsion | 0.13 | 0.72 | 1.42 | 2.42 | Ho | splice site | c.841(+1)G>A | substitution | IVS7 | - |
|  | p4 | 5m | M | convulsion | 0.17 | 0.59 | 1.55 | 2.52 | Ho | nonsense | c.3514C>T | substitution | Exon 25 | - |
|  | p5.1 | 9m | M | Convulsion, mental retradation | 0.21 | 0.62 | 1.68 | 2.25 | Ho | frame shift | c.3158A>G | substitution | Exon23 | + |
|  | P5.2 | 1m | M | Convulsion, specific learning difficulty | 0.16 | 0.51 | 1.82 | 2.37 | Ho | frame shift | c.3158A>G | substitution | Exon23 | + |

P: patient; M: male; F: female; N/A: no applicable; IVS: intervening sequence; CHe: Compound heterozygous; Ho: Homozygous; He: Heterozygou;
